# Estrogen Modulates Glycerol Permeability in Sertoli Cells through Downregulation of Aquaporin-9

**DOI:** 10.3390/cells7100153

**Published:** 2018-09-28

**Authors:** Raquel L. Bernardino, David F. Carrageta, Ana M. Silva, Giuseppe Calamita, Marco G. Alves, Graça Soveral, Pedro F. Oliveira

**Affiliations:** 1 Department of Microscopy, Laboratory of Cell Biology, Institute of Biomedical Sciences Abel Salazar (ICBAS) and Unit for Multidisciplinary Research in Biomedicine (UMIB), University of Porto, 4050-313 Porto, Portugal; raquellbernardino@gmail.com (R.L.B.); davidcarrageta@gmail.com (D.F.C.); anamariapsilva@gmail.com (A.M.S.); alvesmarc@gmail.com (M.G.A.); 2 Department of Biosciences, Biotechnologies and Biopharmaceutics, University of Bari “*Aldo Moro*”, 70125 Bari, Italy; Giuseppe.calamita@uniba.it; 3 Research Institute for Medicines (iMed.ULisboa), Faculty of Pharmacy, Universidade de Lisboa, 1649-003 Lisbon, Portugal; gsoveral@ff.ulisboa.pt; 4 Department of Biochemistry and Human Biology, Faculty of Pharmacy, Universidade de Lisboa, 1649-003 Lisbon, Portugal; 5 Department of Genetics, Faculty of Medicine, University of Porto, 4200-319 Porto, Portugal; 6 i3S-Instituto de Investigação e Inovação em Saúde, University of Porto, 4200-135 Porto, Portugal

**Keywords:** aquaglyceroporins, AQP9, estrogens, glycerol, male infertility, Sertoli cells

## Abstract

High 17β-Estradiol (E2) levels are known to cause alterations of spermatogenesis and environments throughout the male reproductive tract. Sertoli cells (SCs) ensure an adequate environment inside the seminiferous tubule. Glycerol stands as essential for the maintenance of blood–testis barrier created by SCs, however, the role of E2 in this process is not known. Herein, we hypothesized that the effect of E2 on glycerol permeability in mouse SCs (mSCs) could be mediated by aquaglyceroporins. The expression of aquaglyceroporins was assessed by RT-PCR and qRT-PCR. Glycerol permeability was evaluated by stopped-flow light scattering. We were able to identify the expression of AQP3 and AQP9 in mSCs where AQP9 is more abundant than AQP3. Our results show that high E2 levels decrease AQP9 mRNA abundance with no influence on AQP3 in mSCs. Interestingly, high E2 levels decreased mSCs’ permeability to glycerol, while downregulating AQP9 expression, thus suggesting a novel mechanism by which E2 modulates fluid secretion in the testis. In conclusion, E2 is an important regulator of mSCs physiology and secretion through changes in AQP9 expression and function. Thus, alterations in glycerol permeability induced by E2 may be the cause for male infertility in cases associated with the presence of high E2 levels.

## 1. Introduction

In the last two decades, extensive research showed that beyond the classic view on the major relevance of androgens for male reproduction, estrogens can also regulate the development and function of the male reproductive tract. 17β-Estradiol (E2) is found in measurable concentrations in the blood of men and higher concentrations have been reported in the testis and semen [1,2]. E2 is necessary for the healthy development and function of the male reproductive organs. However, elevated levels of this hormone induce deleterious effects that are consistently associated with male subfertility or infertility [2,3]. For instance, elevated E2 levels induce morphological alterations in Sertoli cells (SCs) and Leydig cells that result in severe failures in spermatogenesis [4]. 

High E2 levels change ionic homeostasis and the transport of some molecules within the seminiferous tubule, and some of those molecules may alter or even arrest spermatogenesis. For instance, elevated concentrations of glycerol in testis causes changes in the function of the blood–testis barrier (BTB), compromising the homeostasis of the tubular fluid and leading to the death of germ cells [5]. In fact, while glycerol is essential for spermatogenesis [6], acute exposure to high concentrations of glycerol can temporarily halt spermatogenesis [7]. In addition, chronic exposure to high glycerol concentrations may prompt permanent oligospermia or even azoospermia [5]. SCs are responsible for the maintenance of the BTB [8] and thus for promoting normal spermatogenesis. However, studies concerning SCs’ permeability to glycerol and the expression and function of the associated transport mechanisms are still scarce. 

Aquaporins (AQPs) are a family of channel proteins facilitating the transport of water and a series of small anaelectrolytes across biological membranes [9]. Aquaglyceroporins represent a subgroup of the AQPs family conducting not only water but also small neutral solutes, such as glycerol [10]. Mammalian aquaglyceroporins comprise four known homologues—AQP3, AQP7, AQP9, and AQP10. AQPs are widely located in the male reproductive tract, where they are involved in fluid absorption/secretion dynamics and contribute in maintaining the homeostasis for the occurrence of a normal spermatogenesis [9,11]. Hence, AQPs have emerged as pivotal for healthy male reproductive function and alterations in their expression or function have been suggested to result in subfertility or infertility. Furthermore, there is evidence that estrogens regulate the expression and function of AQPs in efferent ducts and epididymis. It was reported that estrogens modulate water reabsorption through AQP9, which may compromise sperm quality [12,13]. These works led us to envisage that male infertility may be linked to elevated E2 levels and altered expression or function of aquaglyceroporins associated with glycerol permeability in SCs.

Herein, we hypothesized that E2 may regulate glycerol permeability through aquaglyceroporins expression and function in mouse testis, particularly in mouse SCs (mSCs). Thus, the aim of this study was to evaluate the impact of high concentrations of E2 on aquaglyceroporins expression in mSCs, with focus on the expression of AQP3, AQP7, and AQP9 (the *Aqp10* gene is a pseudogene in mouse) [14]. Since these aquaglyceroporins are responsible for the transport of glycerol whose homeostasis is critical for proper male reproductive health, we further evaluated the impact of high E2 on mSCs’ glycerol permeability.

## 2. Material and Methods

### 2.1. Chemicals

NZY Total RNA Isolation kit and NZY M-MuLV Reverse Transcriptase was acquired from NZYtech (Lisboa, Portugal), fetal bovine serum from Biochrom AG (Berlin, German) and all other chemicals were purchased from Sigma-Aldrich (St. Louis, MO, USA) unless stated otherwise. 

### 2.2. Cell Culture and Experimental Groups

Mouse SCs (mSCs), TM4 were purchased from ATCC (Manassas, VA, USA). Cells were seeded in a 75 cm^2^ flask (SPL70075, SPL Life Sciences, Gyeonggi, Korea) in 1:1 mixture of DMEM:F12 supplemented with 1.2 g/L sodium bicarbonate, 50 U/mL penicillin, 50 mg/mL streptomycin sulphate, 0.5 mg/mL fungizone, 50 μg/mL gentamicin, and 5% FBS with physiological concentrations of testosterone (5 µM) and E2 (1 nM).

Cells were grown until reaching a confluence of 70–80%. Then, the culture medium was replaced by phenol-red free DMEM:F12 medium supplemented with ITS (in mg/L: insulin 10, transferrin 5.5, selenium 0.0067; pH 7.4). Cells were separated in two groups, control group and a group treated with high concentration of E2 (100 nM). The E2 concentration was chosen based on published papers, which demonstrated that in intratesticular plasm levels of this hormone are particularly higher than those of circulating plasma, reaching concentrations up to 200 nM [15,16]. The same amount of ethanol (solvent) was used in the cells of the control group that was used in the cells of the E2-treated group (<0.025% *v*/*v*). Treatments were done during 24 h in an atmosphere of 6% CO_2_ and 94% O_2_ at 33 °C. 

### 2.3. Reverse Transcriptase Polymerase Chain Reaction (RT-PCR) and Quantitative RT-PCR (qPCR) 

Extraction of RNA from mSCs and mouse testis was performed using the NZY Total RNA Isolation kit as indicated by the manufacturer. RNA concentrations were determined by Nanodrop 2000 Spectrophotometer (Thermo Fisher Scientific, Waltham, MA, USA). RNA was reversely transcribed using the NZY M-MuLV Reverse Transcriptase. The resultant complementary DNA (cDNA) was used with exon-exon spanning primer sets designed to amplify cDNA fragments described in Table 1. 

Conventional reverse transcriptase PCR (RT-PCR) was performed to identify *Aqp3*, *Aqp7*, and *Aqp9* mRNAs in mSCs and quantitative Real-Time PCR (qPCR) was performed to evaluate the mRNA abundance in cells from the control and E2-treated group as previously described [3]. Briefly, specific primers were designed for the amplification of the *Aqp3*, *Aqp7*, *Aqp9*, and *β-2-microglobulin* transcripts. qPCR conditions were previously optimized and specificity of the amplicons was determined by melting curves. Amplification conditions: 5 min at 95 °C, followed by 30 or 40 runs of a 3 steps cycle: 10 s at 95 °C; 30 s with a specific temperature for each set of primers, and 10 s at 72 °C. β2-microglobulin transcript levels were used to normalize gene expression levels. Fold variation of gene expression levels was calculated following the model proposed by Pfaffl [17], using the formula 2^−ΔΔCt^.

### 2.4. Preparation of Cellular Suspension

mSCs obtained from the control and E2-treated groups were detached with trypsin and centrifuged at 300× *g* (gravitational units) to obtain a cellular pellet. The cells were resuspended in isotonic medium (300 mOsm, in mM: 220 mannitol, 70 sucrose, 20 Tris-HCl, 1 EDTA, 5 EGTA, 1 PMSF, pH 7.4) and left for 10 min to reach the equilibrium in this medium. The cellular preparations were homogeneous and mSCs were spherical in shape when in suspension, as observed under light microscopy. The diameter of cells was measured for all the preparations with ImageJ software with pictures obtained by light microscopy.

### 2.5. Stopped-Flow Light Scattering

Stopped-flow light scattering was performed following an adaptation of the protocol described by Maggio et al. [18] and Campos et al. [19]. Experiments were performed on a HI-TECH Scientific PQ/SF-53 stopped-flow apparatus, which has a 2 ms dead time and is temperature controlled (24 °C), interfaced with an IBM PC/AT compatible 80386 microcomputer. This procedure was performed to measure the membrane permeability of mSCs to glycerol. Osmotic shock was performed with glycerol solution (540 mOsm, in mM: 150 glycerol, 220 mannitol, 70 sucrose, 20 Tris-HCl, 1 EDTA, 5 EGTA, 1 PMSF, pH 7.4). Four runs were usually stored and analysed in each experimental condition. In each run 0.1 mL cellular suspension was mixed with an equal amount of hyperosmotic glycerol solution to reach inwardly directed gradients of solute. After the first fast cell shrinkage due to water outflow, glycerol influx in response to its chemical gradient was followed by water influx with subsequent cell re-swelling. The kinetics of cell re-swelling was measured from the time course of 90° scattered light intensity at 530 nm until a stable light scatter signal was attained. Glycerol permeability (*P*_gly_) was calculated as *P*_gly_ = ki(V_0_/A), where ki is the single exponential time constant (s^−1^) fitted to the light scattering signal of glycerol influx and V_0_/A is the initial cell volume to area ratio. 

Glycerol permeability was measured in mSCs control and E2-treated groups. In addition, both groups were incubated with phloretin, a general inhibitor of aquaglyceroporins [20,21], Phloretin (0.7 mM) was added to the cellular suspension 15 min before permeability measurements, according to previously published data [20,21]. All solution osmolarities were determined from freezing point depression on a semi-micro osmometer (Knauer GmbH, Berlin, Germany) using standards of 100 and 400 mOsm.

### 2.6. Statistical Analysis

Experimental results are presented as mean ± standard error of mean (SEM) (*n* = 6 for each condition, done in triplicate). Statistical analysis was executed using a one-way ANOVA in GraphPad Prism 6 (GraphPad Software, San Diego, CA, USA). *p* < 0.05 was considered significantly different. 

## 3. Results

### 3.1. Aqp3 and Aqp9 are Expressed in mSCs but not AQP7

**Figure 1:**
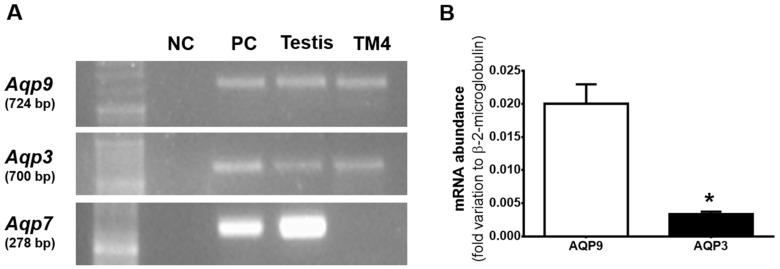
Identification and quantification of mRNA abundance of Aquaporin-3 (*Aqp3*), *Aqp7*, and *Aqp9* in mouse testis and mouse Sertoli cells (mSCs). (**A**) representative reverse transcriptase-PCR experiment. Mouse liver cDNA was used as positive control (PC) for the evaluation of mRNA expression of *Aqp9* and kidney cDNA for *Aqp3* and *Aqp7*. Negative control (NC) was performed without reverse transcriptase enzyme. (**B**) relative abundance of *Aqp9* and *Aqp3* mRNA in mSCs. Results are expressed as mean ± SEM (*n* = 6). * *p* ˂ 0.05.

To date, studies on the role and location of aquaglyceroporins in SCs are virtually nonexistent. In this study, we performed a screen to identify the expression of aquaglyceroporins in mSCs. We were able to identify the expression of *Aqp3* and *Aqp9,* but the presence of *Aqp7* was not detected in mSCs (Figure 1A). The positive control for *Aqp3* and *Aqp7* was mouse kidney and for *Aqp9* was mouse liver. We did not check the fourth aquaglyceroporin, AQP10, since its gene is known to be a pseudogene in mouse [14].

The relative abundance of the *Aqp3* and *Aqp9* transcripts was determined by qRT-PCR, with the primers’ efficiency set to 90–110%. In mSCs, the relative abundance of *Aqp9* (0.0200 ± 0.0029 arbitrary units) resulted about seven times higher than that of *Aqp3* (0.0034 ± 0.0004 arbitrary units) in mSCs (Figure 1B).

### 3.2. High Levels of E2 Downregulate Aqp9 mRNA Expression in mSCs

**Figure 2:**
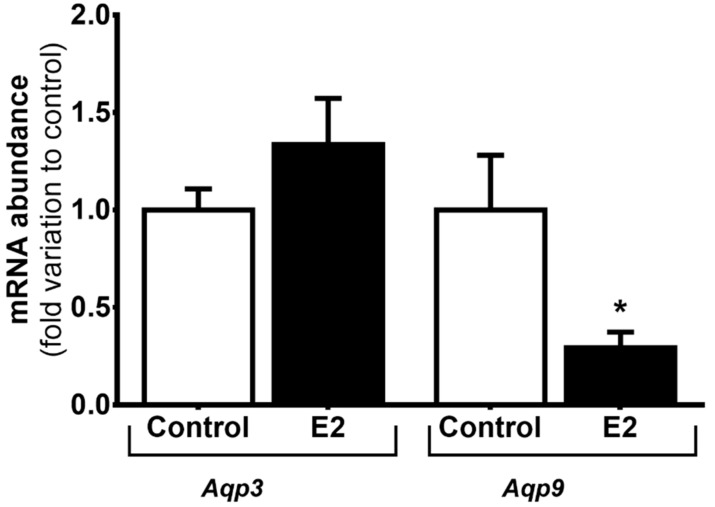
Effect of exposure of mouse Sertoli cells (mSCs) to 17β-estradiol (E2) (100 nM) on Aquaporin-3 (*Aqp3*) and *Aqp9* mRNA abundance. Results are expressed as mean ± SEM (*n* = 6 for each condition). Results are expressed as mean ± SEM (*n* = 6). * *p* ˂ 0.05 relative to control.

We evaluated the mRNA expression of *Aqp3* and *Aqp9* in mSCs treated with high levels of E2 relatively to cells from the control group. The mRNA abundance of *Aqp3* was not altered in cells treated with E2 (1.34 ± 0.24-fold variation to control) in relation to control group (1.00 ± 0.11-fold variation to control). Concerning *Aqp9* mRNA abundance, we observed a significant decrease when mSCs were treated with high E2 levels (0.29 ± 0.08-fold variation to control) in relation to the expression detected in cells from the control group (1.00 ± 0.28-fold variation to control) (Figure 2). 

### 3.3. Permeability of mSCs to Glycerol is Decreased after Exposure to High Levels of E2

**Figure 3:**
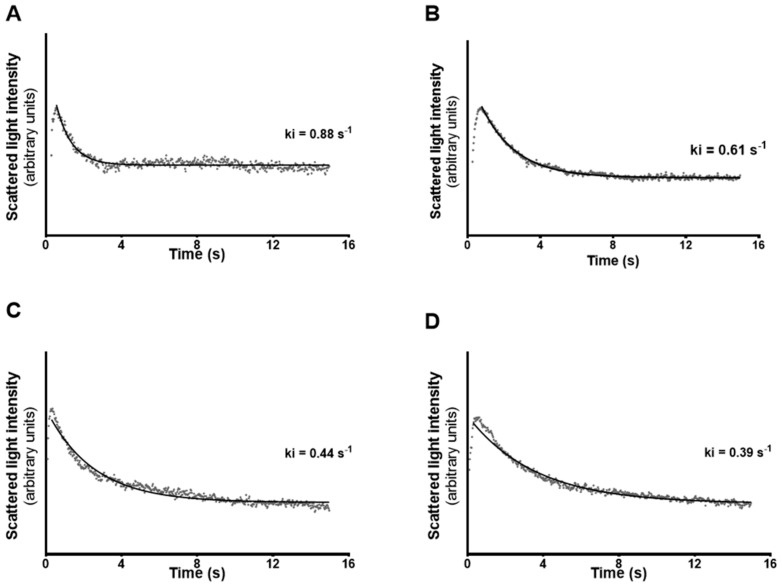
Representative curves of scattering light intensity for glycerol permeability in mouse Sertoli cells (mSCs) under different conditions. (**A**) cells from control group, ki = 0.88 s^−1^; (**B**) cells from group treated with 17β-estradiol (E2) 100 nM, ki = 0.61 s^−1^; (**C**) cells exposed to phloretin (0.7 mM) ki = 0.44 s^−1^; (**D**) cells from group treated with 17β-estradiol (E2) 100 nM and after were exposed to phloretin (0.7 mM), ki = 0.39 s^−1^.

Since aquaglyceroporins allow facilitated diffusion of glycerol across membranes we ran stopped-flow light scattering experiments to evaluate the possible correlation between the expression of the aquaglyceroporins in question and the mSCs membrane permeability. Glycerol permeability (*P*_gly_, µm/s) was calculated by evaluating the diminution in light scattering intensity associated to the osmotic influx of water that followed the entry of glycerol into the mSCs triggered by its inwardly-directed chemical gradient (150 mM; see *Materials and Methods* for details). The diameter of mSCs needed to calculate the *P*_gly_ was measured by optical microscopy before the beginning of the stopped-flow light scattering measurements, when the suspended cells were in osmotic equilibrium with the 300 mOsm solution. The mean cell diameter was 21.50 ± 0.32 µm. Scattered light intensity representative curves of mSCs from control group (A), group exposed to the aquaglyceroporin inhibitor, phloretin (C), E2-treated group (B), and E2-treated group exposed to phloretin (D) are illustrated in Figure 3. 

**Figure 4:**
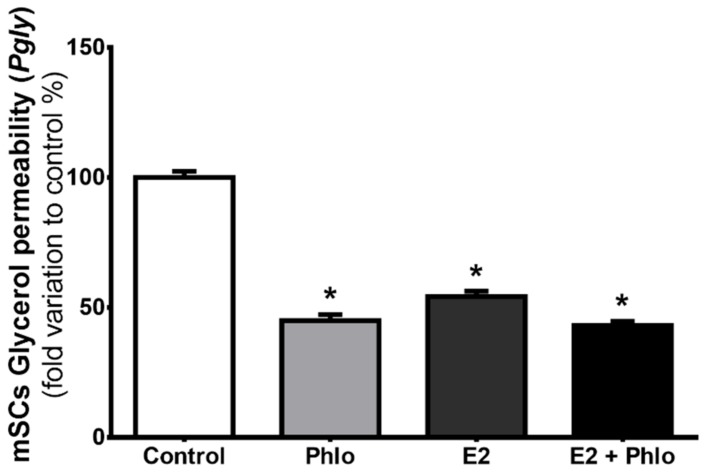
Effects of 0.7 mM phloretin, 17β-estradiol (E2) 100 nM and E2 with exposure to phloretin in mouse Sertoli cells permeability to glycerol (*P*_gly_). Results are expressed as mean ± SEM (*n* = 6). * *p* ˂ 0.05 relative to control.

The inhibition of aquaglyceroporins by phloretin reduced the permeability of mSCs to glycerol by 55% in relation to control (44.92 ± 2.29%, 100 ± 13.38%, respectively). Additionally, exposure to high levels of E2 also significantly reduced the mSCs’ permeability to glycerol, likewise cells treated with E2 and exposed to phloretin (54.21 ± 1.97%, 43.04 ± 1.56%, respectively) (Figure 4). Consistent with the above described aquaglyceroporin downregulation induced by E2, no differences were observed concerning the *P*_gly_ values in mSCs inhibited with phloretin and those exposed with E2 and then treated with phloretin before the glycerol permeability measurements (Figure 4).

## 4. Discussion

The role of estrogens in male reproductive function has been exhaustively investigated in the last decades, providing evidence that estrogens are crucial to the establishment of male reproductive potential [3,23,24]. Fluid reabsorption and ion transport in some regions of the male reproductive tract are known to be regulated by steroid hormones [25,26,27,28]. Indeed, as also reported in liver [29], steroid hormones, namely E2, can modulate the expression of some aquaglyceroporin isoforms and consequently change the fluid reabsorption in efferent ducts and epididymis [23]. For instance, E2 is able to modulate the transcriptional expression of AQP9 in efferent ducts and epididymis. This effect can occur early during sexual development, as exposure of neonatal rats to estrogenic compounds decrease the transcript level of AQP9 in epididymis [13], but these effects were reversed with the administration of testosterone [12]. In contrast, anti-estrogens cause a down-regulation of AQP9 in efferent ducts [30], which may lead to impaired reabsorption in the seminal fluid. Additionally, in the epididymis, it has been reported that orchiectomized rats were devoid of AQP3 in basal epididymal cells. However, when testosterone was administrated, a slightly restoration of AQP3 expression was noted, suggesting that sex steroid hormones can modulate the expression of AQP3 in the epididymis [31]. In fact, similar effects have also been reported for AQP9 expression [25]. Nevertheless, there is contradictory data regarding this subject. There are studies showing that estrogens do not alter AQP9 expression, while androgens seem to modulate AQP9 levels in the initial segment of the epididymis [29], and other where estrogen administration increased AQP9 expression in efferent ducts [13]. These inconsistencies in the literature illustrate the complexity surrounding the regulation of aquaglyceroporins. While there is evidence to support that aquaglyceroporins are under sex steroid hormone regulation, few data are available and further research on the topic is required. Moreover, the expression pattern of aquaglyceroporins throughout the male reproductive tract remains mostly unknown. Thus, we hypothesized that E2 could control the expression of plasma membrane aquaglyceroporins and, consequently, the related cell membrane permeability to glycerol in mSCs, as it happens in other cells and regions of the male reproductive tract.

In this work, we were able to identify, for the first time, the expression of three different aquaglyceroporins transcripts in mouse testis—*Aqp3*, *Aqp7*, and *Aqp9*. Interestingly, the screening in mSCs showed that *Aqp7* is not expressed, but we found presence of the transcripts of two homologues, AQP3 and AQP9, featuring high permeability to glycerol (in addition to other solutes and water). In addition to these aquaglyceroporins, AQP10 and AQP11 are also able to transport glycerol [32,33]. AQP10 is present in the efferent ducts and epididymis in rats [31]. However, AQP10 is a pseudogene in mice and does not encode a functional protein [14]. With regard to AQP11, this transporter was identified in the efferent ducts, epididymis, and testes of rats [34]. In testes, AQP11 is expressed in the elongated spermatids, in the residual bodies, and in the cytoplasm of SCs, being linked with intracellular water and glycerol transport, and regulating organelles volume, but not in the plasma membrane [35]. Thus, as we aimed to evaluate impact of E2 on the expression of cell membrane aquaglyceroporins and on its permeability to glycerol in mSCs, we evaluated the eventual contribution of the three different aquaglyceroporins AQP3, AQP7, andAQP9, but not that of AQP10 and AQP11. There are studies showing that glycerol is essential for the regulation of spermatogenesis and for normal testis morphology [6]. Conversely, intratesticular injection of glycerol in rats resulted in long-term suppression of spermatogenesis and increased permeability in the blood–testis barrier [36]. As BTB is formed by SCs and glycerol induced alterations in the tight junction formed by the union of SCs [5], these “nurse cells” appear to be susceptible to the action of this metabolite and may mediate its negative effect in spermatogenesis. Hence, it is of extreme importance to understand the regulatory mechanisms and dynamics of glycerol permeability since it can define the reproductive potential of males by modulating SCs function. Herein, we studied the mechanisms by which mSCs’ permeability to glycerol occurs and how E2 may modulate it.

Aquaglyceroporins are responsible for the facilitated transport of glycerol across the membranes of the vast majority of cells. Our results showed that mSCs express two isoforms of aquaglyceroporins, *Aqp3* and *Aqp9*. In addition, we observed that *Aqp9* is about seven times more expressed transcriptionally in mSCs than *Aqp3*, indicating that *Aqp9* may have a more relevant role on glycerol and water transport than *Aqp3* in these cells. Permeability studies by stopped-flow light scattering also demonstrated that mSCs permeability to glycerol is inhibited by phloretin in 55% in relation to non-treated cells. Since phloretin is known as a general inhibitor of aquaglyceroporins [22,37,38] and that only two isoforms able to transport glycerol are expressed in mSCs, it is clear that AQP3 and AQP9 are pivotal for the transport of glycerol in the mSCs. Glycerol movement across membranes also occur through the phospholipid bilayer, by “simple diffusion”, a thermodynamically disadvantaged pathway, of less importance and, above all, not controllable with respect to the “facilitated” pathway offered by aquaglyceroporins [20,21].

Previous works have shown that E2 is a regulator of aquaglyceroporins in the male reproductive tract [12,30]. Indeed, as it happens in the epididymis and efferent ducts, aquaglyceroporins are also modulated by E2 in mSCs. However, there was a differential effect concerning the effects on *Aqp3* or *Aqp9*, since high E2 levels downregulated *Aqp9* expression but did not influence *Aqp3* expression. The downregulation of *Aqp9* expression induced by high E2 levels is consistent with the modulation detected in terms of glycerol transport. Through studies using the stopped-flow light scattering technique to assess glycerol permeability in mSCs, we detected a 46% reduction in glycerol permeability after exposure to high levels of E2 in relation to mSCs cultured with physiological concentrations of this hormone. In addition, the inhibition of aquaglyceroporins by phloretin in cells previously treated with high levels of E2 did not cause a significant alteration in glycerol permeability when compared with cells only treated with high levels of E2. Hence, these data suggest that the effect caused by the downregulation of *Aqp9* induced by high levels of E2 is comparable to the inhibitory effect conferred by phloretin on the permeability to glycerol in mSCs. 

Our results show that exposure of mSCs to high levels of E2 causes a decrease in *Aqp9* expression, which leads to decreased glycerol permeability and can lead to dysregulation of the concentration of glycerol in the testis, causing fertility problems or even infertility. Moreover, the number of cases idiopathic infertility associated with increased levels of E2 and high glycerol levels is high [39] and thus, dysfunction of glycerol transport may play a role in those cases. In addition to cases of idiopathic infertility that account for about 30–45% infertility cases due to male factors [40], there are also some known pathologies that are related to hormonal changes in E2 levels and induce male infertility. For instance, Klinefelter syndrome is the most frequent sex chromosome abnormality in men. Men with this syndrome present in most cases low levels of testosterone and increased levels of estrogens and follicle stimulating hormone (FSH) [3]. Most patients affected by this syndrome present azoospermia with consequent infertility [40]. It may be relevant to assess the expression of aquaglyceroporins in SCs and related glycerol permeability in men with Klinefelter’s syndrome. Other disorders, such as obesity, have a great incidence in the world and are steadily increasing. Obesity is regularly associated with hormonal dysregulation [41,42]. In addition, obesity is related with alterations in the expression and function of the aquaglyceroporins and consequently in glycerol transport [43,44]. Obese men are known to have a positive correlation with abnormal sperm morphology, decreased sperm concentration and sperm motility, decreased serum testosterone and increased E2 levels compared with healthy men, which consequently leads to compromised fertility [45,46,47]. In the male reproductive tract, SCs are the principal hormonal targets [48], and since E2 plays such an important role in male reproduction, namely in glycerol permeability by the control of aquaglyceroporins, the study of this cell type is of great importance also to understand the mechanisms by which obesity and metabolic diseases affect the fertility of males.

However, in vitro effects are not always reflected in vivo. Although it is known that glycerol is essential for spermatogenesis, no changes were observed in the reproductive parameters of *Aqp9* null mice [49]. Compensatory effects may be occurring in vivo in this animal model, or the expression/localization of aquaglyceroporins in SCs in vivo may not be exactly the same as in cell cultures. For instance, as in rat testis there is an elevated expression of AQP9 in Leydig cells [50] and these cells strongly influence SCs (namely by the secretion of testosterone), there will be indirect effects on SCs that may be responsible for the reproductive phenotype of *Aqp9* null mice. This is in fact an initial in vitro study, demonstrating that pure cultures of mSCs exposed to high doses of E2 present a decrease in the abundance of *Aqp9* and consequently decrease the permeability to glycerol. In vivo studies evaluating the expression and function of aquaglyceroporins in the testicular tissue of individuals with sex steroid hormones dysregulation (particularly estrogens) will be useful to clarify this matter. In summary, we described, for the first time, the expression of two important glycerol channels in mSCs, AQP3 and AQP9, which may be essential for spermatogenesis due to their ability to mediate the membrane transport of glycerol, a metabolite known to arrest germ cell development. E2 is an essential hormone in the male reproductive tract and we have now reported a new mechanism by which it may impact male fertility: through downregulation of *Aqp9* modulating SCs glycerol permeability. This study may open new insight on the cases of male infertility related to increased E2 levels, which may be associated with an alteration of testicular glycerol levels through changes in *Aqp9* expression, and a consequent negative impact on spermatogenesis. Still, further studies will be needed to correlate these results with an in vivo situation and to highlight a possible association between these mechanisms and idiopathic infertility and other diagnosed cases of male infertility associated with high E2 levels.

## Figures and Tables

**Table 1:** Oligonucleotides and cycling conditions for PCR amplification of *Aquaporin-3* (*Aqp3*), *Aquaporin-7* (*Aqp7*), *Aquaporin-9* (*Aqp9*) and *β-2-microglobulin*. C: Number of cycles.

GENE	SEQUENCE 5′-3′	ANNEALING T°	C
*AQP3* (NM_016689.2)	**FWD**: GGACCCTCATCCTTGTGATGTT **RVS**: TCGTAGTACAGCCCAAAAACAA	63 °C	40
*AQP7* (NM_007473.4)	**FWD**: CTACAGAAGAATATGGTGCGAGA **RVS**: CAGGAACTGACCCAGCACAT	63 °C	40
*AQP9* (NM_022026.3)	**FWD**: CTGAGAAGGACCGAGCCAAG **RVS**: ATGATGACGCTGAGTTCGTGT	60 °C	40
*β-2-MICROGLOBULIN* (NM_009735.3)	**FWD**: GCTTCAGTCGTCAGCATGGC **RVS**: GGATTTCAATGTGAGGCGGGT	58 °C	30
